# Supplementary material for: Polyketides as Secondary Metabolites from the Genus Aspergillus
Source: J Fungi (Basel). 2023 Feb 15;9(2):261. doi: 10.3390/jof9020261 (PMC9962652; doi:10.3390/jof9020261)
Supplement: Supplementary file 1 [file jof-09-00261-s001.zip › jof-2127437-supplementary.pdf]

Supplementary Materials for

Polyketides as Secondary Metabolites from the Genus *Aspergillus*

Xuelian Bai [1], **Yue Sheng** [1], **Zhenxing Tang** [2], **Jingyi Pan** [1], **Shigui Wang** [1], **Bin Tang** [1], **Ting Zhou** [1], **Lu'e Shi** [1], **and Huawei Zhang** [3,*]

[1]College of Life and Environmental Sciences, Hangzhou Normal University, Hangzhou 311121, China
[2]School of Culinary Arts, Tourism College of Zhejiang, Hangzhou 311231, China
[3]School of Pharmaceutical Sciences, Zhejiang University of Technology, Hangzhou 310014, China
[*]Correspondence: hwzhang@zjut.edu.cn; Tel.: +86-571-88320613

Table S1. Detail information for *Aspergillus*-derived polyketides

No.	Name	Strain	Source	Biological activity	Ref.
**1**	pre-Shamixanthone	*A. nidulans* FGSCA4	-	significant inhibitory effect on lipid accumulation in HepG2 cells without cytotoxic effects, accompanying the potent reduction of total cholesterol and triglycerides	[1,2]
**2**	2-(3,5-Dichloro-2,6-dihydroxy-4-methylbenzoyl)-5-hydroxy-3-methoxybenzoic acid	*A. terreus* C9408-3	soil of fumaroles from Yangmingshan Mountain area (China)	-	[3,4]
**3**	Dihydrogeodin	*A. terreus* C9408-3		significant inhibitory effect on the isomerase cyclophilin A.	
**4**	3,5-Dichlorosulochrin	*A. flavipes* PJ03-11	wetland mud	stronger α-glucosidase inhibitory activities than acarbose	[5]
**5**	3-de-O-methylsulochrin				
**6**	2-(3,5-Dichloro-2,6-dihydroxy-4-methylbenzoyl)-5-hydroxy-3-methoxybenzoic acid				
**7**	Eurobenzophenone A	*A. europaeus* WZXY-SX-4-1	marine sponge *Xestospongia testudinaria*	-	[6]
**8**	Eurobenzophenone B			weak inhibitory effects against NO production and the DPPH radical scavenging activity	
**9**	Eurobenzophenone C			potent radical scavenging activity against DPPH	
**10**	14-O-Demethylsulochrin			-	
**11**	3-de-O-methylsulochrin			potent radical scavenging activity against DPPH	
**12**	14-de-Omethyl-5-methoxysulochrin				
**13**	Sulochrin			-	
**14**	5-methoxysulochrin			-	
**15**	Gibellulin C	*A. nidulans* ΔLaeB			[7]
**16**	Gibellulin D				
**17**	F-9775A	*A. nidulans* RMS011	-	strong inhibitory effect on cathepsin K for treatment of osteoporosis	[8]
**18**	F-9775B				
**19**	Tetraorcinol A	*A. versicolor* LCJ-5-4	soft coral *Cladiella* sp. collected from the South China Sea	weak radical-scavenging activity against the DPPH radical with an IC_{50} value of 67 μM	[9]
**20**	Geodin hydrate	*A. terreus* C9408-3	soil of fumaroles from Yangmingshan Mountain area	inhibiory effect on ET-1 binding to the ETA receptor	[3,10]
**21**	Methyl-3,5-dichloroasterric acid			-	
**22**	Asterric acid			inhibiory effect on ET-1 binding to the ETA receptor	
**23**	5-hydroxymethylasterric acid	*A. flavipes* PJ03-11	wetland mud	-	[5]
**24**	Neogeodin hydrate			-	
**25**	2,4-dichloroasterric acid			potent inhibitory effect on α-glucosidase	
**26**	Methyl dichloroasterrate			-	
**27**	Monomethylosoic acid			-	
**28**	Asterric acid			-	
**29**	Methyl 3-chloroasterric acid			-	
**30**	Methyl chloroasterrate			-	

**31**	Diorcinol	*A. flocculus*	stems of the medicinal plant *Markhamia platycalyx*	cytotoxic activity against chronic myelogenous leukemia cell line K562 at 30 µM and inhibition the sleeping sickness causing parasite *Trypanosoma brucei*	[11]
**32**	Asperic acid	*A. niger*	Caribbean sponge, *Hyrtios proteus*	cytotoxic activity against the murine lymphocytic leukemia P388 and a panel of human cancer cell lines	[12,13]
**33**	Asperfuranone	*A. nidulans*	-	cytotoxic activity against A549 cells with an IC_{50} value of 15.3 µM	[14,15]
**34**	2-(20,3-Epoxy-10,30-heptadienyl)-6-hydroxy-5-(3-methyl-2-butenyl)benzaldehyde	*A. glaucus* HB1-19	marine sediment surrounding mangrove roots	strong radical-scavenging activity	[16]
**35**	2-(20,3-Epoxy-10,30,50-heptatrienyl)-6-hydroxy-5-(3-methyl2-butenyl)benzaldehyde				
**36**	2,3,4-trimethyl-5,7-dihydroxy2,3-dihydrobenzofuran	*A. terreus* X3	soils collected from Sichuan	no antimicrobial effect	[17]
**37**	Flufuran	*A. flavus* 9643	-	inhibit the phytopathogen Phytophthora cinnamomi at 0.2 mg/mL	[18,19]
**38**	3,7-dihydroxy-1,9-dimethyldibenzofuran	*A. sydowii* SCSIO 41301	marine sponge *Phakellia fusca*	-	[20,21]
**39**	Asperochratide H	*A. ochraceus*	deep-sea water of the Northeastern Pacific Ocean	significant cytotoxic effect on BV-2 cell line	[22]
**40**	Asperpentenone A	*Aspergillus* sp. SCSIO 41024	deep-sea sample in the South China Sea	-	[23]
**41**	Asticolorin A	*A. versicolor* MRC 638	-	-	[24,25]
**42**	Asticolorin B				
**43**	Asticolorin C				
**44**	Penicillic acid	*A. ochraceus*	marine sponge	antibacterial effect on phytopathogens	[26,27]
**45**	Versicolactone A	*A. versicolor* LCJ-5-4	soft coral *Cladiella* sp. collected from the South China Sea		[9,28]
**46**	Versicolactone B			excellent cytotoxicity against human pancreatic cancer cells with an IC_{50} values of 9.4 µM	
**47**	Carlosic acid	*A. niger* ATCC1015	-	-	[29]
**48**	Agglomerin F			-	
**49**	Carlosic acid methyl ether			-	
**50**	2-carboxymethyl-3-hexylmaleic acid anhydride	*A. tubingensis* OY907	Mediterranean marine sponge *Ircinia variabilis*	inhibitory effect on *Neurospora crassa* (MIC = 207 µM)	[30]
**51**	Aspiketolactonol	*Aspergillus* sp. 16-02-1	deep-sea sediment sample collected at Lau Basin hydrothermal vent in southwest Pacific	cytotoxic activity against human cancer K562, HL-60, HeLa, and BGC-823 cell lines	[31]
**52**	Aspilactonol A				
**53**	Aspilactonol B				
**54**	Aspilactonol C				
**55**	Aspilactonol D				

**56**	Aspilactonol E		Ocean		
**57**	Aspilactonol F				
**58**	(*S*)-2-(2′-hydroxyethyl)-4-methyl-*γ*-butyrolactone				
**59**	Asperochrin B	*A. ochraceus* MA-15	rhizospheric soil of marine mangrove plant *Bruguiera gymnorrhiza*	-	[32]
**60**	Penicillic acid			antibacterial activity against *Aeromonas hydrophilia*, *Vibrio anguillarum*, and *V. harveyi* with IC_{50} values ranging from 0.5 to 32.0 μg/mL	
**61**	Chlorohydroasperlactone A				
**62**	Chlorohydroasperlactone B			-	
**63**	Aspergone A	*Aspergillus* sp. OUCMDZ-1583	marine sponge XD10410 from the Xisha Islands (China)	inhibitory effect on *α*-glucosidase with an IC_{50} value of 2.36 mM	[33]
**64**	Aspergone B			inhibitory effect on *α*-glucosidase with an IC_{50} value of 1.65 mM	
**65**	Aspergone C			-	
**66**	Aspergone D			-	
**67**	Dihydropenicillic acid	*A. flocculus*	stems of the medicinal plant *Markhamia platycalyx*	-	[11]
**68**	Asperochratide F	*A. ochraceus*	deep-sea water of the Northeastern Pacific Ocean	strong cytotoxic effects on BV-2 cell line	[22]
**69**	Gregation B	*A. flavus*	-	antibacterial activity against *E. coli* ATCC 25922	[18,34]
**70**	Avenaciolide	*A. avenaceus* G. Smith	-	inhibitory effect on the transport of glutamate in rat liver mitochondria and the ability of ADP to stimulate the rate of glutamate oxidation	[35]
**71**	Citrifuran A	*Aspergillus* sp.	intestines of centipedes	moderate inhibitory activities against LPS-induced NO production in RAW 264.7 macrophages with IC_{50} values of 18.3, 22.6, and 25.3 μM, respectively	[36]
**72**	Citrifuran B				
**73**	Citrifuran C				
**74**	Citrifuran D			-	
**75**	Asperone A			-	[37]
**76**	Asperone B			showed significant nitric oxide (NO) inhibition in lipopolysaccharide (LPS)-induced RAW 264.7 macrophage cells, and exhibited IC_{50} values of 16.0 μM.	
**77**	Asperlactone	*A. melleus* CMI 49108	-	inhibitory effect on superoxide anion	[38,39]
**78**	Chlorocarolide A	*A. ochraceus*	marine sponge	-	[36]
**79**	Chlorocarolide B			-	
**80**	Protulactone A	*Aspergillus* sp. SF-5044	inter-tidal sediment sample collected from the Dadaepo Beach, Busan, (Korea)	-	[40]
**81**	Tubingenoic anhydride A	*A. tubingensis* OY907	marine sponge *Ircinia*	inhibitory effect on *Neurospora crassa* growth with a MIC	[30]

			variabilis	value of 330 μM	
**82**	Asperochrin A	*A. ochraceus* MA-15	rhizospheric soil of marine mangrove plant *Bruguiera gymnorrhiza*.	antibacterial activity against *Aeromonas hydrophila*, *Vibrio anguillarum* and *V. harveyi*	[38]
**83**	Aspergone E	*Aspergillus* sp. OUCMDZ-1583	marine sponge XD10410 from the Xisha Islands (China)	inhibitory effect on *α*-glucosidase with an IC_{50} value of 1.30 mM	[33]
**84**	Aspergone F			-	
**85**	Allahabadolactone A	*A. allahabadii* BCC45335	root of *Cinnamomum subavenium* Miq. at Khao Yai National Park (Thailand)	cytotoxicity against NCI-H187 and Vero cell lines with IC_{50} values of 17.78 and 31.50 μg/mL, respectively	[41]
**86**	Allahabadolactone B			anti-*B. cereus* with an IC_{50} value of 12.50 μg/mL and cytotoxicity against NCI-H187 and Vero cell lines with IC_{50} values of 30.51 and 21.00 μg/mL, respectively	
**87**	Asperone C	*Aspergillus* sp.	intestines of centipedes	significant nitric oxide (NO) inhibition in lipopolysaccharide (LPS)-induced RAW 264.7 macrophage cells	[37]
**88**	Asperone D				
**89**	Asperone E			-	
**90**	Asperochratide A	*A. ochraceus*	deep-sea water of the Northeastern Pacific Ocean	-	[22]
**91**	Asperochratide B			-	
**92**	Asperochratide C			-	
**93**	Asperochratide D			-	
**94**	Asperochratide E			strong cytotoxic effect on BV-2 cell line	
**95**	Asperochratide F			-	
**96**	(+)-geodin	*P. glabrum* AJ117540<br>*A. terreus* ATCC 20542	-	stimulated glucose uptake by rat adipocytes	[42,43]
**97**	Asperetide	*Aspergillus* sp. TJ23	leaves of the traditional Chinese plant *Hypericum perforatum* Linn.	-	[44]
**98**	(5)-3-butyl-7-methoxyphthalide				
**99**	Porriolide	*A. nidulans*	-	inhibitory effect on the root elongation of both lettuce and stone-leek seedlings by 53.3% and 48.5%, respectively antifungal activities against *Fusarium graminearum*, *Botrytis cinerea* and *Phytophthora nicotianae* with MIC values of 3.1, 25 and 6.3 μg/mL, respectively	[7,45,46]
**100**	3-methoxyporriolide			-	
**101**	7-methoxyporriolide			-	
**102**	(-)-(R)-mellein	*A. ochraceus*	Indo-Pacific sponge *Jaspis coriacea*	a broad spectrum of antifungal and antioomycetes activities	[49]
**103**	Botryoisocoumarin A (a racemate)	*A. ochraceus* MA-15	rhizospheric soil of marine mangrove plant *Bruguiera*	-	[32]
**104**	(3*R*,4*S*)-4-hydroxymellein			-	
**105**	(3*R*,4*R*)-4-hydroxymellein			-	

**106**	(R)-7-hydroxymellein		*gymnorrhiza*	antibacterial activity against aquatic pathogens *Aeromonas hydrophila*, *Vibrio anguillarum* and *V. harveyi*	
**107**	6-O-demethylmonocerin	A*spergillus* sp. OUCMDZ-1583	marine sponge XD10410 from the Xisha Islands (China)	inhibitory effect on α-glucosidase with an IC_{50} value of 0.027 mM and influenza A (H1N1) virus with an IC_{50} value of 172.4 µM	[33]
**108**	7-O-demethylmonocerin			-	
**109**	(+)-monocerin			inhibitory effect on α-glucosidase with an IC_{50} value of 1.65 mM and influenza A (H1N1) virus with an IC_{50} value of 175.5 µM	
**110**	fusarentin 6-methyl ether			inhibitory effect on α-glucosidase with an IC_{50} value of 1.19 mM	
**111**	6,7-O-dimethyl-4R-hydroxy-10-epifusarentin			inhibitory effect on α-glucosidase with an IC_{50} value of 1.74 mM	
**112**	(3*S*)-5-hydroxymellein	*Aspergillus* sp. SCSIO XWS03F03	marine sponge collected from the sea area Xuwen County, Guangdong Province (China)	-	[50,51]
**113**	Aflatoxin B1	*A. flavus*	-	strong toxicity	[18,52,53]
**114**	Aflatoxin G1				
**115**	Aflatoxin B2		-		
**116**	Botryoisocoumarin A	*A. flocculus*	stems of the medicinal plant *Markhamia platycalyx*	inhibited the growth of chronic myelogenous leukemia cell line K562 at 30 µM	[11]
**117**	Mullein			-	
**118**	cis-4-hydroxymellein			-	
**119**	trans-4-hydroxymellein			-	
**120**	3-hydroxymellein			exhibited a respective inhibition of 56% to the sleeping sickness causing parasite *Trypanosoma brucei*	
**121**	5-hydroxymellein			inhibited the growth of chronic myelogenous leukemia cell line K562 at 30 µM	
**122**	4,5-dihydroxymellein			-	
**123**	(*S*)-6,8-dimethoxy-3-methylisochroman-1-one	*A. terreus* SCSIO 41008	Marine sponge *Callyspongia* sp.	exhibited no cytotoxic activities towards human glioma U87 cells at a concentration of 10 µmol/L and showed no protective activity against glutamate-induced toxicity in HT22 cells at a concentration of 10 µmol/L	[54]
**124**	Alternariol 9-O-methyl ether	*A. fumigatus* D	coastal plant *Edgeworthia chrysantha* Lindl.	no antimicrobial activity	[55]
**125**	Asperjinone	*A. terreus* C9408-3	soil of fumaroles from Yangmingshan	-	[3,56-58]
**126**	Butyrolactone I			mild cytotoxic activity	

**127**	Butyrolactone IV		Mountain area (China)		
**128**	Butyrolactone V			antiplasmodial activity against *Plasmodium falciparum* K1 with an IC_{50} value of 7.9 µg/mL	
**129**	Aspulvinone P	*A. flavipes* PJ03-11	wetland mud	stronger *α*-glucosidase inhibitory effect than acarbose	[5]
**130**	Aspulvinone Q				
**131**	MethybutyrolactoneIII				
**132**	Flavipes in B				
**133**	Butyrolactone II				
**134**	Microperfuranone	*A. nidulans*	-	-	[10,59]
**135**	Aspergillolide	*A. carneus* L03	fresh and healthy potato tissue collected from Lincang, Yunnan Province, China	moderate antifungal activity against plant pathogens and inhibitory effect on nitric oxide production in lipopolysaccharide-stimulated RAW264.7 cells with IC_{50} values of 13.36 and 30.16 µM, respectively.	[60]
**136**	(±)-asperteretal D				
**137**	Isotorachrysone	*A. glaucus*	marine sediments surrounding mangrove roots collected in the Fujian Province, (China)	no cytotoxicity at 100 µM against the HL-60 and A-549 cell lines	[61]
**138**	Isotorachrysone-6-O-R-D-ribofuranoside				
**139**	8-methoxy-3-methyl-1-naphthalenol-6-O-R-D-ribofuranoside				
**140**	8-methoxy-1-naphthalenol-6-O-α-R-D-ribofuranoside				
**141**	Asperflavin				
**142**	Isoasperflavin				
**143**	Neosartoricin B	*A. nidulans* RJMP1.49	-	-	[62]
**144**	Neosartoricin C			-	
**145**	Neosartoricin D			-	
**146**	Funalenone	*A. niger* FGSC A1279	-	inhibitory effect on types I and IV collagenases	[63]
**147**	Methyl 6-acetyl-4methoxy-5,7,8-trihydroxynaphthalene-2-carboxylate	*A. terreus* SCSIO 41008.	marine sponge *Callyspongia* sp.	weak or no cytotoxic activities towards human glioma U87 cells and weak or no protective activity against glutamate-induced toxicity in HT22 cells	[54]
**148**	Methyl 6-acetyl-4-methoxy-5, 8-dihydroxynaphthalene-2-carboxy -late				
**149**	Orsellinic acid	*A. nidulans* RMS011	-	cytotoxic activity toward HepG2 and CCF cell lines	[64,65]
**150**	Lecanoric acid			-	
**151**	2,4-Dihydroxy-6-((R)-4-hydroxy-2-oxopentyl)-3methylbenzaldehyde	*A. versicolor*	marine sponge *Petrosia* sp.	no cytotoxicity against cell lines A-549, SK-OV-3, SK-MEL-2, XF498 or HCT-15	[66]
**152**	Aspergentisyl A	*A. glaucus* HB1-19	marine sediment surrounding mangrove roots	strong radical-scavenging activity	[16]
**153**	Aspergentisyl B				
**154**	Dihydroauroglaucin				
**155**	Tetrahydroauroglaucin				
**156**	Isodihydroauroglaucin				
**157**	Flavoglaucin				
**158**	Auroglaucin				
**159**	Flavipin	*A. fumigatus* AF3-093A	the brown alga *Fucus*	antimicrobial effect on *S. aureus*, methicillin-resistant *S.*	[67]

			vesiculosus	*aureus* and *Mycobacterium tuberculosis* H37Ra	
**160**	Porosuphenol A	*A. porosus* G23	Bioviotica	no activity was observed for antifungal, antimalaria, antitubercular, antioxidant, and metal-chelating activity (>50 μM).	[68]
**161**	Porosuphenol B				
**162**	(**a**) Porosuphenol C<br>(**b**) Porosuphenol D				
**163**	Hydroxysydonic acid	*A. flavus* 9643	-	inhibitory effect on NO production in LPS-stimulated BV2 cells with IC_{50} values in 76.5 μM	[69,70]
**164**	Versicolorin B	*Aspergillus* sp. F40	marine sponge *Callyspongia* sp.	moderate activities against *S. aureus* and methicillin-resistant *S. aureus*	[71,72]
**165**	Methyl 2-(4-hydroxyphenyl) acetate	*A. flocculus*	stems of the medicinal plant *Markhamia platycalyx*	-	[11]
**166**	(**a**) 2-hydroxyphenyl acetic acid<br>(**b**) 4-hydroxyphenyl acetic acid			-	
**167**	*p*-hydroxybenzaldehyde			-	
**168**	Aspergillusene D	*A. sydowii* SCSIO 41301	marine sponge *Phakellia fusca*	-	[21,73,74]
**169**	3-(2,5-Dimethylbenzo[*d*][1,3]dioxol-2-yl)propanoic acid				
**170**	2-(5-Hydroxy-4-methylpentyl)-2-methylbenzo[*d*][1,3]dioxole-5-<br>carboxylic acid				
**171**	Sydonic acid			-	
**172**	(*E*)-7-deoxy-7,8-didehydrosydonic acid			moderate anti-*C. capsici*, *C. gloeosporioides*, and *A. brassicicola* effects with MIC values of 12.5, 12.5, 25 mg/mL, respectively	
**173**	(*Z*)-7-deoxy-7,8-didehydrosydonic acid			-	
**174**	7-deoxy-7,14-didehydrosydonic acid			-	
**175**	(+)-12hydroxysydonic acid			-	
**176**	3,4-dihydroxy-phenylacetic acid methyl ester	*Aspergillus* sp. SCSIO 41024	deep-sea sample in the South China Sea	strong free-radical-scavenging activity	[23,75]
**177**	Fumagillin	*Aspergillus* sp.	-	antibacterial activity against *Staphylococcus aureus*	[76]
**178**	Aspinonene	*A. ochraceus* FH-A6692	-	-	[77]
**179**	Aspinotriol A	*A. ostianus* 01F313	an unidentified marine sponge collected in Pohnpei	cytotoxic effects on cell lines K562, HL-60, HeLa and BGC-823	[44,78]
**180**	Aspinotriol B				
**181**	Aspinonediol			-	
**182**	Epiaspinonediol	*Aspergillus* sp. 16-02-1	deep-sea sediment sample collected at Lau Basin hydrothermal vent in southwest Pacific Ocean	exhibited weak antitumor effect on K562, HL-60, HeLa, and BGC-823 cell lines but no anti-MRSA activity	
**183**	Aspergone I	*Aspergillus* sp. OUCMDZ-1583	marine sponge XD10410 from the	-	[33]
**184**	Aspergone J			inhibitiory effect on *α*-glucosidase with an IC_{50} value of 2.37	

			Xisha Islands (China)	mM	
**185**	Aspergone K			inhibitiory effect on *α*-glucosidase with an IC_{50} value of 2.70 mM	
**186**	Aspergone L			-	
**187**	Aspergone M			-	
**188**	(-) palitantin	*A. fumigatiaffnis*	endophytic fungus from a medicinal plant *Tribulus terestris*	antimicrobial effect on multi-resistant clinical isolates of *Enterococcus faecalis* and *Streptococcus pneumoniae* with a MIC value of 64 µg/mL.	[79]
**189**	Azanigerone A	*A. niger* ATCC 1015	Agricultural Research Service (ARS) culture collection	-	[80]
**190**	Azanigerone B				
**191**	Azanigerone C				
**192**	Azanigerone E				
**193**	Azanigerone F				
**194**	Citrinin	*A. terreus* ATCC 20542	-	protective effect on iron-induced lipid peroxidation and neuroprotective property	[81]
**195**	3*R*,4*S*-3,8-Dimethoxy-3-methylisochromane-4,6-diol	*A. fumigatus*	endophytic fungus isolated from *Cordyceps sinensis*	inhibitory activity against the MV4-11 cell line *in vitro* with an IC_{50} value of 23.95 µM	[82]
**196**	3*R*,4*R*-3,8-Dimethoxy-3-methylisochromane-4,6-diol			-	
**197**	3*S*,4*R*-3,8-Dimethoxy-3-methylisochromane-4,6-diol			inhibitory activity against the MV4-11 cell line *in vitro* with an IC_{50} value of 32.70 µM	
**198**	3*R*,4*S*-3,8-Dimethoxy-3-methylisochromane-4,6-diol			-	
**199**	3,6,8-Trimethoxy-3-methylisochromane			-	
**200**	Asperochratide I	*A. ochraceus*	deep-sea water of the Northeastern Pacific Ocean	no cytotoxic, anti-food allergic, anti-H1N1 virus and anti-inflammatory activities	[22]
**201**	Asperochratide J				
**202**	Protulactone B	*Aspergillus* sp. SF-5044	inter-tidal sediment sample collected from the Dadaepo Beach, Busan, (Korea)	-	[40]
**203**	Chaetoquadrin F	*Aspergillus* sp. 16-02-1	deep-sea sediment sample collected at Lau Basin hydrothermal vent in southwest Pacific Ocean	antitumor activity against HeLa with IR% values 13.5% at 100 µg/mL	[31]
**204**	Asperochrin C	*A. ochraceus* MA-15	rhizospheric soil of marine mangrove plant *Bruguiera gymnorrhiza*	-	[32]
**205**	Chlorohydroaspyrone A			inhibitory activity against aquatic pathogenic bacterial *Aeromonas hydrophila*, *Vibrio anguillarum*, and *V. harveyi*.	
**206**	Chlorohydroaspyrone B				
**207**	Dihydroaspyrone			-	
**208**	Aspyronol			-	

**209**	12,13-Dihydroxymagnaporthepyrone	*A.oryzae* M-2-3	rice blast fungus	-	[83]
**210**	10,11-Dihydroxymagnaporthepyrone			-	
**211**	(+)-asperlin	*A.nidulans* mutant	-	anti-inflammatory, antitumor and antibiotic activity	[84]
**212**	Nigerpyrone	*A. niger* FGSC A1279	-	-	[85]
**213**	Aspopyrone A	*Aspergillus* sp. TMPU1623	a root part of an Okinawan plant	strong inhibitory effect on protein tyrosine phosphatase (PTP) 1B with an IC_{50} value of 6.7 μM	[86]
**214**	Dihydroaspyrone	*A. flocculus*	stem of the medicinal plant *Markhamia platycalyx*	-	[11]
**215**	Kojic acid			weak antibacterial activities against *Bacillus thuringiensis* and *Acinetobacter baumanni*i with MIC values of 32 and 128 μg/mL, respectively	
**216**	7-O-acetyl kojic acid			-	
**217**	4-hydroxy-3,6-dimethyl-2pyrone	*A. sydowii* SCSIO 41301	marine sponge *Phakellia fusca*	-	[21]
**218**	4-methyl-5,6-dihydropyren-2-one				
**219**	Phomapyrone C	*Aspergillus* sp. SCSIO 41024	deep-sea sediments in the South China Sea	-	[23]
**220**	Aspergchromone A	*Aspergillus* sp. SCSIO XWS03F03	marine sponge collected from the sea area Xuwen County, Guangdong Province (China)	-	[50]
**221**	Aspergchromone B				
**222**	Noreugenin				
**223**	Aurasperone A	*A. niger* FGSC A1279	-	toxic activity against brine shrimp with a LD_{50} value of 9 ppm	[85,87]
**224**	Fonsecinone D			-	
**225**	Rubrofusarin B	*A. fumigatus* D	coastal plant *Edgeworthia chrysantha* Lindl.	no potent antimicrobial activity against *E. coli*, *S.aureus* and *C. albicans*	[55]
**226**	Asperpyrone A				
**227**	Asperpyrone D				
**228**	Fonsecinone B				
**229**	Fonsecinone A				
**230**	Aspergiolide A	*A. glaucus* HB1-19	marine sediment surrounding mangrove roots	selective cytotoxicities against A-549, HL-60, BEL-7402, and P388 cell lines	[61,88,89]
**231**	Aspergiolide B			potent cytotoxicities against the HL-60 and A-549 cell lines with IC_{50} values of 0.51 and 0.24 μM, respectively.	
**232**	Emodin			-	
**233**	Physcion			-	
**234**	Questin			-	
**235**	Catenarin			-	
**236**	Rubrocristin			-	
**237**	Physcion bianthrones			-	

**238**	(*trans*)-emodin-physcion bianthrone			moderate cytotoxicities against HL-60 and A-549 cell lines with IC_{50} values of 7.8 and 9.2 µM, respectively.	
**239**	(*cis*)-emodin-physcion bianthrone			cytotoxic effect on HL-60 and A-549 cell lines with IC_{50} values 44.0 and 14.2 µM, respectively	
**240**	(+)-variecolorquinone A			-	
**241**	Aspergiolide C			strong inhibitory effect on tyrosine kinases (RTKs) c-Met, Ron, and c-Src with low-micromolar IC_{50}s	
**242**	Aspergiolide D				
**243**	Averantin	*A. versicolor*	marine sponge *Petrosia* sp.	significant cytotoxicity against tumor cell lines (A-549, SK-OV-3, SK-MEL-2, XF-498, and HCT-15) with IC_{50} values in the range of 0.41-4.61 µg/mL	[66]
**244**	Methyl-averantin				
**245**	Averufin			-	
**246**	Nidurufin			antibacterial activity against clinically isolated Gram-positive strains with MIC values of 6.25 µg/mL.	
**247**	Versiconol			weak antimicrobial activity against *V. parahaemolyticus* with a MIC value of 12 µg/mL.	
**248**	Sanghaspirodin A	*A. nidulans*	-	moderate antibiotic activity against Gram-positive bacteria and good antiproliferative effect on human leukaemia and endothelial as well as cytotoxicity against human cervix carcinoma cell lines	[90]
**249**	Sanghaspirodin B				
**250**	2ω-dihydroxyemodin	*A. nidulans* FGSCA4	-	-	[91]
**251**	Chrysophanol			-	
**252**	ω-hydroxyemodin-5-methyl ether	*A. terreus* C9408-3	soil of fumaroles from Yangmingshan Mountain (China)	-	[3]
**253**	ω-acetylcarviolin			-	
**254**	Dermolutein	*A. europaeus* WZXY-SX-4-1	marine sponge *Xestospongia testudinaria*	remarked down-regulation of NF-κB in LPS-induced SW480 cells with weak inhibitory effects against NO production and the DPPH radical scavenging activity	[6]
**255**	Methylemodin				
**256**	(+)1-O-demethylvariecolorquinones A			-	
**257**	1-methoxy-14-dehydroxywentiquinone C			-	
**258**	Wentiquinone C			-	
**259**	Asperthecin	*A. nidulans*	-	potent aggregation inhibitory effect	[92]
**260**	Versiconol B	*Aspergillus* sp. F40	marine sponge *Callyspongia* sp.	weak antimicrobial activity against *S. aureus* and *V. parahaemolyticus* with MIC values of 48 µg/mL and 24 µg/mL, respectively.	[71]
**261**	Dihydrosterigmatocystin			-	
**262**	Secosterigmatocystin			-	
**263**	1,6,8-trihydroxy-3-methylanthraquinone	*A. sydowii* SCSIO 41301	marine sponge *Phakellia fusca*	-	[21]
**264**	Emodic acid			broad inhibitory activities against three influenza A virus subtypes A/Puerto Rico/8/34 (H1N1), A/Aichi/2/68 (H3N2) and A/ FM-1/1/47 (H1N1), respectively	
**265**	Parietinic acid				

**266**	1,8-dihydroxy-3-methoxy-6-methylanthracene-9,10-dione	*A. terreus* SCSIO 41008	marine sponge *Callyspongia* sp.	weak or no cytotoxic activities towards human glioma U87 cells and weak or no protective activity against glutamate-induced toxicity in HT22 cells at 10 μM	[54]
**267**	1-methyl emodin				
**268**	8-di-O-methylaverufin	*A. versicolor* HBU-2017-7	Bohai Sea (China)	-	[93]
**269**	1'-O-methylaverantin				
**270**	Spinulosin	*A. flavus*	-	effective nematicidal activity against *B. xylophilus* without any plant growth inhibition	[18,94,95]
**271**	Terreic acid	*A. terreus* ATCC 20542	-	inhibotry effect on Bruton's tyrosine kinase	[42,96]
**272**	Phomaligol A	*A. flocculus*	stem of the medicinal plant *Markhamia platycalyx*	-	[11]
**273**	Phomaligol A1			moderate activity against *T. brucei* with a MIC value of 25 μg/mL	
**274**	Csypyrone B1	*A. oryzae* M-2-3	-	-	[97]
**275**	Aspergiodiquinone	*A. glaucus* HB1-19	marine sediment surrounding mangrove roots	-	[16]
**276**	(4*S*)-6-hydroxyisosclerone	*Aspergillus* sp. SCSIO XWS03F03	sponge collected from the sea area Xuwen County, Guangdong Province(China)	-	[50,98]
**277**	(-)-regiolone			no significant cytotoxicity against human tumor cell lines HeLa, A549 and HepG2	
**278**	Ergosterol	*Aspergillus* sp. TJ23	fresh leaves of the traditional Chinese plant *Hypericum perforatum* L	inhibitory activities against five cancer cell lines (B16, MDA-MB-231, 4Tl, HepG2, and LLC) with IC_{50} values ranging from 5.13 to 12.3 μM.	[48]
**279**	Ergosterol peroxide	*A. flocculus*	Stem of the medicinal plant *Markhamia platycalyx*	inhibitory effect on the migration of MDA-MB-231 cells at concentrations lower than 20 μM.	[11,99,100]
**280**	Campesterol			-	
**281**	steroid	*Aspergillus* sp. SCSIO 41017	deep-sea sample in the South China Sea	moderate activity against brain cancer cell line (SF-268), breast cancer cell line (MCF-7), human liver cancer cell line (HepG-2) and human lung carcinoma cell line (A549) with IC_{50} values of 13.5-18.0 μM	[101]
**282**	Terretonin	*A. terreus*	-	-	[102]
**283**	Fumicycline A	Cocultivation of a strain of *A. fumigatus* with the actinomycete *Streptomyces rapamycinicus*	the soil-derived bacterium*Streptomyces rapamycinicus*and the human pathogenic fungus *Aspergillus fumigatus*	-	[103]
**284**	Fumicycline B			-	
**285**	Parasiticolide A	*A. flavus* and *A. parasiticus* IFO 4082	-	-	[18,104]
**286**	Spiroaspertrione A	*Aspergillus* sp. TJ23	fresh leaves of the	antibacterial toward MRSA with a MIC value of 4 μg/mL	[105]

**287**	Aspermerodione		traditional Chinese plant *Hypericum perforatum* L	potential inhibitor of PBP2a and synergistically with the *β*-lactam antibiotics oxacillin and piperacillin against MRSA.	[106]
**288**	Andiconin C			weak antimicrobial activity	
**289**	Sphaeropsidin A	*A. porosus* G23	marine-derived endophyte	selective cytotoxicity toward melanoma and kidney cancer cell lines with a unique mechanism of action targeting regulatory volume increase.	[68,107]
**290**	Aspergiloid E			-	
**291**	Arugosin C	*A. versicolor* HBU-2017-7	Bohai Sea (China)	-	[93,108]
**292**	Chlovalicin	*A. niger* BRF-074	sediments collected in the Northeast coast of Brazil	no cytotoxicity towards the HCT-116 cell line	[109]
**293**	Sterigmatocystin	*A. versicolor*	marine sponge *Petrosia* sp.	significant cytotoxicity against five human solid tumor cell lines (A-549, SK-OV-3, SK-MEL-2, XF-498, and HCT-15) with IC_{50} values in the range of 0.41-4.61 µg/mL.	[66,71]
**294**	Dihydrosterigmatocystin			-	
**295**	5-methoxysterigmatocystin	*A. versicolor* HBU-2017-7	Bohai Sea (China)	inhibitory effect on *Staphylococcus aureus*, *Bacillus subtilis*, *Pseudomonas aeruginosa*, *Escherichia coli*, and *Candida albicans* is weak.	[93]
**296**	Shamixanthone	*A. nidulans* FGSCA4	-	moderate activities and was selective against gastric carcinoma, colon carcinoma, and breast carcinoma.	[110]
**297**	Secalonic acid D	*A. Aculeatus*<br>*A.* sp. SCSIO XWS03F03	unidentified sponge<br>-	moderate antimicrobial activity against *Staphyloccocus aureus* ATCC 29213 and *Mycobacterium tuberculosis* with IC_{50} values of 7.19 and 1.26 µM, respectively.	[50,111]
**298**	Secalonic acid F	*A. Aculeatus*<br>*A. aculeatus* IBT 21030	-	-	
**299**	Penicitrinone A	*A. terreus* X3	soil fungus collected from Sichuan (China)	moderate activity against Gram-positive bacterium *B. megaterium* with a MIC value of 1.60 µM and moderate activity against Gram-negative bacterium *V. parahemolyticus* with a MIC value of 25.0 µM.	[17]
**300**	Penicitrinone B			moderate activity against Gram-negative bacterium *Vibrio anguillarum* with a MIC value of 12.5 µM	
**301**	Asperanthone	*Aspergillus* sp. TJ23	fresh leaves of the traditional Chinese plant *Hypericum perforatum* Linn.	inhibitory activity against HepG2 with an IC_{50} value of 35.5 µM	[44]
**302**	Ruguloxanthone C			inhibitory activities against the growth of B16, HepG2, and LLC cancer cell lines with IC_{50} values of 35.6, 29.5 and 32.7 µM, respectively	
**303**	Tajixanthone hydrate			inhibitory activity against HepG2 with an IC_{50} value of 36.8 µM	
**304**	Tajixanthone methanoate			-	

**305**	Euroxanthone A	*A. europaeus* WZXY-SX-4-1	marine sponge *Xestospongia testudinaria*	strong down-regulation of NF-κB in LPS-induced SW480 cells with weak inhibitory effects against NO production and the DPPH radical scavenging activity	[6,71]
**306**	Euroxanthone B			-	
**307**	Calyxanthone			-	
**308**	Yicathin C			-	
**309**	Yicathin A			-	
**310**	Yicathin B			strong down-regulation of NF-κB in LPS-induced SW480 cells with weak inhibitory effects against NO production and the DPPH radical scavenging activity	
**311**	Oxisterigmatocystin I	*Aspergillus* sp. F40	sponge *Callyspongia* sp.	-	[71]
**312**	Oxisterigmatocystin C			weak antimicrobial activity against *S. aureus* with an MIC value of 48 μg/mL	
**313**	Sydowic acid			-	
**314**	Averufin			-	
**315**	2-Hydroxy-1-(hydroxymethyl)-8-methoxy-3-methyl-9H-xanthen-9-one	*A. sydowii* SCSIO 41301	sponge *Phakelliafusca*	selective inhibitory activities against two influenza A virus subtypes, A/Puerto Rico/8/34 (H1N1) and A/FM-1/1/47 (H1N1), with IC50 values ranging from 2.17 ± 1.39 to 4.70 ± 1.11 μM	[21]
**316**	2-Hydroxy-1-(hydroxymethyl)-7,8-dimethoxy-3-methyl-9H-xanthen-9-one				
**317**	Mevinolin	*A. terreus* ATCC 20542	soil isolation program at the CEPA Laboratories	potent inhibitory effect on hydroxymethylglutaryl-coenzyme A reductase	[112]
**318**	Mevinolinic acid			-	
**319**	Aspermytin A	*Aspergillus* sp.	marine mussel, *Mytilus edulis*	significant neurotrophic effect on the rat pheochromocytoma (PC-12) cells	[113]
**320**	Decumbenone C	*A. sulphureus* KMM 4640	marine sediments	significant cytotoxicity against melanoma cell lines but not against colorectal cancer	[114, 115]
**321**	Decumbenone A				
**322**	Decumbenone B				
**323**	Calbistrin A	*A. aculeatus* IBT 21030	-	antifungal activity, nerve growth factor-increasing and cholesterol-lowering effects	[116,117]
**324**	Calbistrin C			-	
**325**	Versicorin	*A. versicolor* SC0156	a soil sample collected in the Dinghu Mountain Biosphere Reserve, Guangdong (China)	no bacterial inhibition at 50 μM or cytotoxicity at 100 μM	[118]
**326**	Versiol				
**327**	Aspergone N	*Aspergillus* sp. OUCMDZ-1583	marine sponge XD10410 from the Xisha Islands of	*α*-glucosidase inhibition with an IC_{50} value of 1.36 mM.	[33,44]
**328**	Aspergone O			*α*-glucosidase inhibition with an IC_{50} value of 1.54 mM.	
**329**	Aspergone P			*α*-glucosidase inhibition with an IC_{50} value of 2.21 mM.	

**330**	Aspergone Q		China	α-glucosidase inhibition with an IC_{50} value of 2.26 mM.	
**331**	Epoxyquinol			-	
**332**	Salimyxin B	*A.* sp. TJ23	endophytic fungus isolated from the medicinal plant Hypericum perforatum L	cytotoxic effec on HepG2 with an IC_{50} value of 9.87 µM.	[44]
**333**	Hexylitaconic acid	*A. niger*	Caribbean sponge, *Hyrtios proteus*	inhibitory effect on p53–HDM2 interaction, and antibacterial activities against *E. coli*, *Micrococcus luteus*, *Pseudomonas agarici* and *S. warneri* with MIC values ranging between 0.29 and 0.58 µg/mL.	[29, 119,120]
**334**	Terrain-α-D-glucoside	*Aspergillus* sp. PF1381	soil sample collected in Hachijo Island, Tokyo, Japan	inhibited angiogenin secretion from androgendependent prostate cancer cell line LNCaP-CR with an IC_{50} value of 13 µM	[121]
**335**	Aspercyclide A	*Aspergillus* sp. MF6215	soil sample collected in Olduvai Gorge	inhibitory effect on the *IgE* binding to its receptor with an IC_{50} value of 200 µM.	[122]
**336**	Aspercyclide B			-	
**337**	Aspercyclide C				
**338**	Aculene C	*A. aculeatus* IBT 21030	-	weak or no activity against *Candida albicans*.	[116]
**339**	Aculene D				
**340**	Dehydrocurvularin	*A. terreus* ATCC 20542	-	anti-cancer activity in a glioma stem cell-based orthotopic xenograft model in mice	[47,123,124]
**341**	Aspergone G	*Aspergillus* sp. OUCMDZ-1583	marine sponge XD10410 collected from Xisha Islands (China)	-	[33]
**342**	Aspergone H				
**343**	Terrain	*A. flavus*	-	strong cytotoxicity against breast cancer MCF-7 cells	[3,18]

References

1. Sarkara, A.; Funka, A.N.; Scherlach, K.; Horn, F.; Schroeckh, V.; Chankhamjon, P.; Westermann, M.; Roth, M.; Brakhage, A.A.; Hertweck, C.; et al. Differential expression of silent polyketide biosynthesis gene clusters in chemostat cultures of *Aspergillus* nidulans. *J. Biotechnol.* **2012**, 160, 64-71. [10.1016/j.jbiotec.2012.01.015]
2. Wu, Q.; Wu, C.M.; Long, H.L.; Chen, R.; Liu, D.; Proksch, P.; Guo, P.; Lin, W.H. Varioxiranols A-G and 19-o-methyl-22-methoxypre-shamixanthone, PKS and hybrid PKS-derived metabolites from a sponge-associated *Emericella variecolor* fungus. *J. Nat. Prod.* **2015**, 78, 2461-2470. [10.1021/acs.jnatprod.5b00578]
3. Liao, W.Y.; Shen, C.N.; Lin, L.H.; Yang, Y.L.; Han, H.Y.; Chen, J.W.; Kuo, S.C.; Wu, S.H.; Liaw, C.C. Asperjinone, a nor-neolignan, and terrein, a suppressor of ABCG2-expressing breast cancer cells, from thermophilic *Aspergillus terreus*. *J. Nat. Prod.* **2012**, 75, 630-635. [10.1021/np200866z]
4. Hamed, A.; Ismail, M.; Shaaban, M. X-ray, structural assignment and molecular docking study of dihydrogeodin from *Aspergillus* terreus TM8. *Nat. Prod. Res.* **2019**, 33, 117-121. [10.1080/14786419.2018.1431642]

5. Zhang, L.H.; Feng, B.M.; Zhao, Y.Q.; Sun, Y.; Liu, B.; Liu, F.; Chen, G.; Bai, J.; Hua, H.M.; Wang, H.F. Polyketide butenolide, diphenyl ether, and benzophenone derivatives from the fungus *Aspergillus flavipes* PJ03-11. *Bioorg. Med. Chem. Lett.* **2016**, 26, 346-350. [10.1016/j.bmcl.2015.12.009]
6. Du, X.W.; Liu, D.; Huang, J.; Zhang, C.J.; Proksch, P.; Lin, W.H. Polyketide derivatives from the sponge associated fungus *Aspergillus europaeus* with antioxidant and NO inhibitory activities. *Fitoterapia* **2018**, 130, 190-197. [10.1016/j.fitote.2018.08.030]
7. Lin, H.; Lyu, H.N.; Zhou, S.; Yu, J.W.; Keller, N.P.; Chen, L.; Yin, W.B. Deletion of a global regulator *LaeB* leads to the discovery of novel polyketides in *Aspergillus nidulans. Org. Biomol. Chem.* **2018**, 16, 4973-4976. [10.1039/c8ob01326h]
8. Schroeckh, V.; Scherlach, K.; Nutzmann, H.W.; Shelest, E.; Schmidt-Heck, W.; Schuemann, J.; Martin, K.; Hertweck, C.; Brakhage, A.A. Intimate bacterial-fungal interaction triggers biosynthesis of archetypal polyketides in *Aspergillus nidulans*. *Proc. Natl. Acad. Sci. U S A.* **2009**, 106, 14558-14563. [10.1073/pnas.0901870106]
9. Zhuang, Y.B.; Teng, X.C.; Wang, Y.; Liu, P.P.; Wang, H.; Li, J.; Li, G.Q.; Zhu, W.M. Cyclopeptides and polyketides from coral-associated fungus, *Aspergillus versicolor* LCJ-5-4. *Tetrahedron* **2011**, 67, 7085-7089. [10.1016/j.tet.2011.07.003]
10. Ohashi, H.; Akiyama, H.; Nishikori, K.; Mochizuki, J. Asterric acid, a new endothelin binding inhibitor. *J. Antibiot.* **1992**, 45, 1684-1685. [10.7164/antibiotics.45.1684]
11. Tawfike, A.F.; Romli, M.; Clements, C.; Abbott, G.; Young, L.; Schumacher, M.; Diederich, M.; Farag, M.; Edrada-Ebel, R. Isolation of anticancer and anti-trypanosome secondary metabolites from the endophytic fungus *Aspergillus flocculus* via bioactivity guided isolation and MS based metabolomics. *J. Chromatogr. B.* **2019**, 1106-1107, 71-83. [10.1016/j.jchromb.2018. 12.032]
12. Varoglu, M.; Crews, P. Biosynthetically diverse compounds from a saltwater culture of sponge-derived *Aspergillus niger*. *J. Nat. Prod.* **2000**, 63, 41-43. [10.1021/np9902892]
13. Pettit, G.R.; Du, J.; Pettit, R.K.; Knight, J.C.; Doubek, D.L. Antineoplastic agents. 575. the fungus *Aspergillus phoenicis*. *Heterocycles* **2009**, 79, 909-916. [10.3987/COM-08-S(D)63]
14. Chiang, Y.M.; Szewczyk, E.; Davidson, A.D.; Keller, N.; Oakley, B.R.; Wang, C.C.C. A gene cluster containing two fungal polyketide synthases encodes the biosynthetic pathway for a polyketide, asperfuranone, in *Aspergillus nidulans*. *J. Am. Chem. Soc.* **2009**, 131, 2965-2970. [10.1021/ja8088185]
15. Wang, C.C.C; Chiang, Y.M.; Praseuth, M.B.; Kuo, P.L.; Liang, H.L.; Hsu, Y.L. Asperfuranone from *Aspergillus nidulans* inhibits proliferation of human non-small cell lung cancer A549 cells via blocking cell cycle progression and inducing apoptosis. *Basic Clin. Pharmacol* . **2010**, 107, 583-589. [10.1111/j.1742-7843.2010.00545.x]
16. Sun, S.W.; Ji, C.Z.; Gu, Q.Q.; Li, D.H.; Zhut, T.J. Three new polyketides from marine-derived fungus *Aspergillus glaucus* HB1-19. *J. Asian Nat. Prod. Res.* **2013**, 15, 956-961.[10.1080/10286020.2013.826205]
17. Xu, L.L.; Cao, F.; Tian, S.S.; Zhu, H.J. Alkaloids and polyketides from the soil fungus *Aspergillus terreus* and their antibacterial activities. *Chem. Nat. Compd+.* **2017**, 53, 1212-1215. [10.1007/s10600-017-2243-5]
18. Saldan, N.C.; Almeida, R.T.R.; Avincola, A.; Porto, C.; Galuch, M.B.; Magon, T.F.S.; Pilau, E.J.; Svidzinski, T.I.E.; Oliveira, C.C. Development of an analytical method for identification of *Aspergillus flavus* based on chemical markers using *HPLC-MS. Food Chem.* **2018**, 241, 113-121. [10.1016/j.foodchem.2017.08.065]
19. Evidente, A.; Cristinzio, G.; Punzo, B.; Andolfi, A.; Testa, A.; Melck, D. Flufuran, an antifungal 3,5-disubstituted furan produced by *Aspergillus flavus* link. *Chem. Biodivers.* **2009**, 6, 328-334. [10.1002/cbdv.200800292]
20. Tanahashi, T.; Takenaka, Y.; Nagakura, N.; Hamada, N. Dibenzofurans from the cultured lichen mycobionts of *Lecanora cinereocarnea*. *Phytochemistry* **2001**, 58, 1129-1134. [10.1016/S0031-9422(01)00394-6]
21. Liu, N.Z.; Peng, S.; Yang, J.; Cong, Z.W.; Lin, X.P.; Liao, S.R.; Yang, B.; Zhou, X.F.; Zhou, X.J.; Liu, Y.H.; et al. Structurally diverse sesquiterpenoids and polyketides from a sponge-associated fungus *Aspergillus* sydowii SCSIO41301. *Fitoterapia* **2019**, 135, 27-32. [10.1016/j.fitote.2019.03.031]
22. Zou, Z.B.; Zou, Z.B.; Zhang, G.; Li, S.M.; He, Z.H.; Yan, Q.X.; Lin, Y.K.; Xie, C.L.; Xia, J.M.; Luo, Z.H.; Luo, L.Z.; et al. Asperochratides A-J, Ten new polyketides from the deep-sea-derived *Aspergillus ochraceus*. *Bioorg. Chem.* **2020**, 105, 104349. [10.1016/j.bioorg.2020.104349]
23. Chen, W.H.; Liu, H.Y.; Long, J.Y.; Tao, H.M.; Lin, X.P.; Liao, S.R.; Yang, B.; Zhou, X.F.; Liu, Y.H.; Wang, J.F.; et al. Asperpentenone A, a novel polyketide isolated from the deep-sea derived fungus *Aspergillus* sp. SCSIO 41024. *Phytochem. Lett.* **2020**, 35, 99-102. [10.1016/j.phytol.2019.11.009]

24. Steyn, P.S.; Vleggaar, R.; Simpson, T.J. Stable isotope labelling studies on the biosynthesis of asticolorin C by *Aspergillus multicolor*. Evidence for a symmetrical intermediate. *J. Chem. Soc., Chem. Commun.* **1984**, 12, 765-767.[10.1039/C39840000765]
25. Rabie, C.J.; Simpson, T.J.; Steyn, P.S.; van Rooyen, P.H.; Vleggaar, R. Structure and absolute configuration of the asticolorins, toxic metabolites from *Aspergillus* multicolor. *J. Chem. Soc., Chem. Commun.* **1984**, 12, 764-765.[10.1039/C39840000764]
26. Abrell, L.M.; Borgeson, B. ; Crews, P. Chloro polyketides from the cultured fungus (*Aspergillus*) separated from a marine sponge. *Tetrahedron Lett.* **1996**, 37, 2331-2334. [10.1016/0040-4039(96)00277-8]
27. Nguyen, H.T.; Yu, N.H.; Jeon, S.J.; Lee, H.W.; Bae, C.H.; Yeo, J.H.; Lee, H.B.; Kim, I.S.; Park, H.W.; Kim, J.C. Antibacterial activities of penicillic acid isolated from *Aspergillus persii* against various plant pathogenic bacteria. *Lett. Appl. Microbiol.* **2016**, 62, 488-493. [10.1111/lam.12578]
28. Qi, C.X.; Gao, W.X.; Guan, D.Y.Z.; Wang, J.P.; Liu, M.T.; Chen, C.M.; Zhu, H.C.; Zhou, Y.; Lai, Y.J.; Hu, Z.X.; et al. Butenolides from a marine-derived fungus *Aspergillus terreus* with antitumor activities against pancreatic ductal adenocarcinoma cells. *Bioorg. Med. Chem.* **2018**, 26, 5903-5910. [10.1016/j.bmc.2018.10.040]
29. Yang, X.L.; Awakawa, T.; Wakimoto, T.; Abe, I. Three acyltetronic acid derivatives: noncanonical cryptic polyketides from *Aspergillus niger* identified by genome mining. *Chembiochem*. **2014**, 15, 1578-1583. [10.1002/cbic.201402172]
30. Koch, L.; Lodin, A.; Herold, I.; Ilan, M.; Carmeli, S.; Yarden, O. Sensitivity of *Neurospora crassa* to a marine-derived *Aspergillus tubingensis* anhydride exhibiting antifungal activity that is mediated by the MAS1 protein. *Mar. Drugs* **2014**, 12, 4713-4731. [10.3390/md12094713]
31. Chen, X.W.; Li, C.W.; Cui, C.B.; Hua, W.; Zhu, T.J.; Gu, Q.Q. Nine new and five known polyketides derived from a deep sea-sourced *Aspergillus* sp. 16-02-1. *Mar. Drugs* **2014**, 12, 3116-3137. [10.3390/md12063116]
32. Liu, Y.; Li, X.M.; Meng, L.H.; Wang, B.G. Polyketides from the marine mangrove-derived fungus *Aspergillus ochraceus* MA-15 and their activity against aquatic pathogenic bacteria. *Phytochem. Lett*. **2015**, 12, 232-236. [10.1016/j.phytol.2015.04.009]
33. Kong, F.D.;Zhao, C.Y; Hao, J.J.; Wang,C.; Wang,W.; Huang,X.L.; Zhu, W.M. New α-glucosidase inhibitors from a marine sponge-derived fungus, *Aspergillus* sp. OUCMDZ-1583. *RSC Adv.* **2015**, 5, 68852-68863.[10.1039/C5RA11185D]
34. Wijeratne, E.M.K.; Xu, Y.M.; Arnold, A.E.; Gunatilaka, A.A.L. Pulvinulin A, Graminin C, and cis-Gregatin B – new natural furanones from *pulvinula* sp. 11120, a fungal endophyte of cupressus arizonica. *Nat. Prod. Commun.* **2015**, 10, 107-111.[10.1177/1934578X1501000127]
35. Castelo-branco, P.A.; Rubinger, M.M.M.; Alves, L.D.C.; de Barros, P.M.; Pereira, S.G.; de Melo, V.J.; Pilo-Veloso, D.; Zambolim, L. Synthesis and antifungal activity of aromatic bis-gamma-lactones analogous to avenaciolide. *Chem. Biodivers* **2007**, 4, 2745-2754.[10.1002/cbdv.200790223]
36. Yin, G.P.; Wu, Y.R.; Yang, M.H.; Li, T.X.; Wang, X.B.; Zhou, M.M.; Lei, J.L.; Kong, L.Y. Citrifurans A-D, four dimeric aromatic polyketides with new carbon skeletons from the fungus *Aspergillus* sp. *Org. Lett.* **2017**, 19, 4058-4061. [10.1021/acs.orglett. 7b01823]
37. Yin, G.P.; Wu, Y.R.; Han, C.; Wang, X.B.; Gao, H.L.; Yin, Y.; Kong, L.Y.; Yang, M.H. Asperones A–E, five dimeric polyketides with new carbon skeletons from the fungus *Aspergillus* sp AWG 1–15. *Org. Chem. Front.* **2018**, 5, 2432-2436. [10.1039/c8qo00070k]
38. Garson, M.J.; Staunton, J.; Jones, P.G. New polyketide metabolites from *Aspergillus melleus*: structural and stereochemical studies. *J. Chem. Soc. Perk T1* **1984**, 1,1021-1026. [10.1039/P19840001021]
39. Sakhri, A.; Chaouche, N.K.; Catania, M.R.; Ritieni, A.; Santini, A. Chemical composition of *Aspergillus* creber extract and evaluation of its antimicrobial and antioxidant activities. *Pol. J. Microbiol.* **2019**, 68, 309-316. [10.33073/pjm-2019-033]
40. Sohn, J.H.; Oh, H.C. Protulactones A and B: two new polyketides from the marine-derived fungus *Aspergillus* sp. SF-5044. *B Korean Chem. Soc.* **2010**, 31, 1695-1698. [10.5012/bkcs.2010.31.6.1695]
41. Sadorn, K.; Saepua, S.; Boonyuen, N.; Laksanacharoen, P.; Rachtawee, P.; Prabpai, S.; Kongsaeree, P.; Pittayakhajonwut, P. Allahabadolactones A and B from the endophytic fungus, *Aspergillus allahabadii* BCC45335. *Tetrahedron* **2016**, 72, 489-495. [10.1016/j.tet.2015.11.056]

42. Boruta, T.; M. Bizukojc. Culture-based and sequence-based insights into biosynthesis of secondary metabolites by *Aspergillus terreus* ATCC 20542. *J. Biotechnol.* **2014**, 175, 53-62.[10.1016/j.jbiotec.2014.01.038]
43. Sato, S.; Okusa, N.; Ogawa, A.; Ikenoue, T.; Seki, T.; Tsuji, T. Identification and preliminary SAR studies of (+)-geodin as a glucose uptake stimulator for rat adipocytes. *J. Antibiot. (Tokyo)* **2005**, 58, 583-589. [10.1038/ja.2005.79]
44. Qiao, Y.B.; Tu, K.; Feng, W.Y.; Liu, J.J.; Xu, Q.Q.; Tao, L.; Zhu, H.C.; Chen, C.M.; Wang, J.P.; Xue, Y.B.; et al. Polyketide and prenylxanthone derivatives from the endophytic fungus *Aspergillus* sp. TJ23. *Chem. Biodivers.* **2018**, 15, e1800395. [10.1002/cbdv.201800395]
45. Suemitsu, R.; Ohnishi, K.; Horiuchi, M.; Morikawa, Y.; Sakaki, Y.; Matsumoto, Y. Structure of porriolide, a new metabolite from *Alternaria porri*. *Biosci. Biotech. Biochem.* **1993**, 57, 334-335. [10.1271/bbb.57.334]
46. Yang, X.L.; Zhang, S.; Hu, Q.B.; Luo, D.Q.; Zhang, Y. Phthalide derivatives with antifungal activities against the plant pathogens isolated from the liquid culture of *Pestalotiopsis photiniae*. *J. Antibiot .(Tokyo)* **2011**, 64, 723-727. [10.1038/ja.2011.82]
47. Pochet, L.; Frederick, R.; Masereel, B. Coumarin and isocoumarin as serine protease inhibitors. *Curr. Pharm. Design* **2004**, 10, 3781-3796. [10.2174/1381612043382684]
48. Hussain, H.; Green, I. A patent review of two fruitful decades (1997-2016) of isocoumarin research. *Expert Opin. Ther. Pat.* **2017,** 27, 1267-1275. [10.1080/13543776.2017.1344220]
49. Cimmino, A.; Maddau, L.; Masi, M.; Linaldeddu, B.T.; Evidente, A. Secondary metabolites produced by *Sardiniella urbana*, a new emerging pathogen on European hackberry. *Nat. Prod. Res.* **2019**, 33, 1862-1869. [10.1080/14786419.2018.1477154]
50. Wang, Y.; Lin, X.P.; Ju, Z.R.; Liao, X.J.; Huang, X.J.; Zhang, C.; Zhao, B.X.; Xu, S.H. Aspergchromones A and B, two new polyketides from the marine sponge-associated fungus *Aspergillus* sp. SCSIO XWS03F03. *J. Asian Nat. Prod. Res.* **2017**, 19, 684-690. [10.1080/10286020.2016.1231673]
51. Bi, Y.; Bi, X.; Zhao, Q.; Fang, A.; Chen, Y. Dihydroisocoumarins from the fungus *Cephalosporium* sp. AL031. *Pol. J. Chem.* **2006**, 37, 397-401.[10.1002/chin.200630211]
52. Chang, S.B.; ABDEL-KADER, M.M.; WICK, E.L.; WOGAN, G.N. Aflatoxin B2: chemical identity and biological activity. *Science* **1963**, 142, 1191-1192. [10.1126/science.142.3596.1191]
53. Klich, M.A. *Aspergillus flavus*: the major producer of aflatoxin. *Mol. Plant Pathol.* **2007**, 8, 713-722.[10.1111/j.1364-3703.2007. 00436.x]
54. Luo, X.W.; Lin, Y.; Lu, Y.J.; Zhou, X.F.; Liu, Y.H. Peptides and polyketides isolated from the marine sponge-derived fungus *Aspergillus terreus* SCSIO 41008. *Chin. J. Nat. Medicines* **2019**, 17, 149-154. [10.1016/S1875-5364(19)30017-2]
55. Hua, Y.; Pan, R.; Bai, X.L.; Wei, B.; Chen, J.W.; Wang, H.; Zhang, H.W. Aromatic polyketides from a symbiotic strain *Aspergillus fumigatus* D and characterization of their biosynthetic gene D8.t287. *Mar. Drugs* **2020**, 18,324. [10.3390/md18060324]
56. Rao, K.V.; Sadhukhan, A.K. ; Veerender, M. ; Ravikumar, V. ; Mohan, E.V.S.; Dhanvantri, S.D. ; Sitaramkumar, M . ; Babu, J.M. ; Vyas, K.; Reddy, G.O. Butyrolactones from *Aspergillus terreus*. *Chem. Pharm. Bull. (Tokyo)* **2000**, 48, 559-562.[10.1248/cpb.48.559]
57. Lin, T.; Lu, C.; Shen, Y. Secondary metabolites of *Aspergillus* sp. F1, a commensal fungal strain of Trewia nudiflora. *Nat. Prod. Res.* **2009**, 23, 77-85. [10.1080/14786410701852826]
58. Haritakun, R.; Rachtawee, P.; Chanthaket, R.; Boonyuen, N.; Isaka, M. Butyrolactones from the fungus *Aspergillus terreus* BCC 4651. *Chem. Pharm. Bull. (Tokyo)* **2010**, 58, 1545-1548.[10.1248/cpb.58.1545]
59. Furukawa, T.; Fukuda, T.; Nagai, K.; Uchida, R.; Tomoda, H. Helvafuranone produced by the fungus *Aspergillus nidulans* BF0142 isolated from hot spring-derived soil. *Nat. Prod. Commun.* **2016**, 11,1001-1003.[10.1177/1934578X1601100733]
60. Zhang, X.; Zhang, F.L.; Wu, X.; Ye, K.; Lv, X.; Ai, H.L.; Liu, J.K. Bioactive polyketides from the potato endophytic fungus *Aspergillus carneus*. *Nat. Prod. Commun.* **2020**, 15,1-5. [10.1177/1934578X20985222]

61. Du, L.; Liu, H.B.; Fang, Y.C.; Zhu, W.M.; Gu, Q.Q. Cytotoxic polyketides from a marine-derived fungus *Aspergillus glaucus*. *J. Nat. Prod.* **2008**, 71, 1837-1842. [10.1021/np800303t]
62. Yin, W.B.; Chooi, Y.H.; Smith, A.R.; Cacho, R.A.; Hu, Y.C.; White, T.C.; Tang, Y. Discovery of cryptic polyketide metabolites from dermatophytes using heterologous expression in *Aspergillus nidulans*. *ACS Synth. Biol.* **2013**, 2, 629-634. [10.1021/sb4000 48b]
63. Inokoshi, J.; Shiomi, K.; Masuma, R.; Tanaka, H.; Yamada, H.; Omura, S. Funalenone, a novel collagenase inhibitor produced by *Aspergillus niger*. *J. Antibiot. (Tokyo)* **1999**, 52, 1095-1100. [10.7164/antibiotics.52.1095]
64. Lunne, F.; Niehaus, E.M.; Lipinski, S.; Kunigkeit, J.; Kalinina, S.A.; Humpf, H.U. Identification of the polyketide synthase PKS7 responsible for the production of lecanoric acid and ethyl lecanorate in *Claviceps purpurea*. *Fungal Genet. Biol.* **2020**, 145, 103481. [10.1016/j.fgb.2020.103481]
65. Bogo, D.; Matos, M.D.C.; Honda, N.K.; Pontes, E.C.; Oguma, P.M.; Santos, E.C.D.; de Carvalho, J.E.; Nomizo, A. In vitro antitumour activity of orsellinates. *Z Naturforsch. C J. Biosci.* **2010**, 65, 43-48.[10.1515/znc-2010-1-208]
66. Lee, Y.M.; Li, H.; Hong, J.; Cho, H.Y.; Bae, K.S.; Kim, M.A.; Kim, D.K.; Jung, J.H. Bioactive metabolites from the sponge-derived fungus *Aspergillus versicolor*. *Arch. Pharm. Res.* **2010**, 33, 231-235. [10.1007/s12272-010-0207-4]
67. Flewelling, A.J.; Bishop, A.I.; Johnson, J.A.; Gray, C.A. Polyketides from an endophytic *Aspergillus fumigatus* isolate inhibit the growth of mycobacterium tuberculosis and MRSA. *Nat. Prod. Commun.* **2015**, 10,1661-1662.[10.1177/1934578X1501001009]
68. Neuhaus, G.F.; Adpressa, D.A.; Bruhn, T.; Loesgen, S. Polyketides from marine-derived *Aspergillus* porosus: challenges and opportunities for determining absolute configuration. *J. Nat. Prod.* **2019**, 82, 2780-2789. [10.1021/acs.jnatprod.9b00416]
69. Hamasaki, T.; Nagayama, K.; Hatsuda, Y. Two new metabolites, sydonic acid and hydroxysydonic acid, from *Aspergillus sydowi*. *Agri. Biol. Chem.* **1978**, 42, 37-40.[10.1080/00021369.1978.10862919]
70. Quang, T.H.; Phong, N.V.; Hanh, T.T.H.; Cuong, N.X.; Ngan, N.T.T.; Oh, H.; Nam, N.H.; Minh, C.V. Cytotoxic and immunomodulatory phenol derivatives from a marine sponge-derived fungus *Ascomycota* sp. VK12. *Nat. Prod. Res.* **2021**,35,5153-5159. [10.1080/14786419.2020.1786829]
71. Tian, Y.Q.; Lin, S.T.; Kumaravel, K.; Zhou, H.; Wang, S.Y.; Liu, Y.H. Polyketide-derived metabolites from the sponge-derived fungus *Aspergillus* sp. F40. *Phytochem. Lett.* **2018**, 27, 74-77. [10.1016/j.phytol.2018.06.009]
72. Hu, J.S.; Li, Z.; Gao, J.Y.; He, H.T.; Dai, H.Q.; Xia, X.K.; Liu, C.H.; Zhang, L.X.; Song, F.H. New diketopiperazines from a marine-derived fungus strain *Aspergillus versicolor* MF180151. *Mar. Drugs* **2019**, 17,e262. [10.3390/md17050262]
73. Kudo, S.; Murakami, T.; Miyanishi, J.; Tanaka, K.; Takada, N.; Hashimoto, M. Isolation and absolute stereochemistry of optically active sydonic acid from *Glonium* sp. (Hysteriales, Ascomycota). *Biosci. Biotech. Bioch.* **2009**, 73, 203-204. [10.1271/bbb.80535]
74. Wei, M.Y.; Wang, C.Y.; Liu, Q.A.; Shao, C.L.; She, Z.G.; Lin, Y.C. Five sesquiterpenoids from a marine-derived fungus *Aspergillus* sp. isolated from a gorgonian Dichotella gemmacea. *Mar. Drugs* **2010**, 8, 941-949. [10.3390/md8040941]
75. Zhang, Z.Z.; Xiao, B.H.; Chen, Q.; Lian, X.Y. Synthesis and biological evaluation of caffeic acid 3,4-dihydroxyphenethyl ester. *J. Nat. Prod.* **2010**, 73, 252-254. [10.1021/np900519d]
76. Hanson, F.R.; Eble, T.E. An antiphage agent isolated from *Aspergillus Sp. J. Bacteriol.* **1949**, 58, 527-529. [10.1128/JB.58.4.527-529.1 949]
77. Fuchser, J.; Grabley, S.; Noltemeyer, M.; Philipps, S.; Thiericke, R.; Zeeck, A. Secondary metabolites by chemical-screening .28. aspinonene, a new multifunctional fungal metabolite. *Liebigs Annalen Der Chemie* **1994**, 8,831-835.[10.1002/jlac.199419940812]
78. Kito, K.; Ookura, R.; Yoshida, S.; Namikoshi, M.; Ooi, T.; Kusumi, T. Pentaketides relating to aspinonene and dihydroaspyrone from a marine-derived fungus, *Aspergillus ostianus*. *J. Nat. Prod.* **2007**,70, 2022-2025. [10.1021/np070301n]

79. Ola, A.R.B.; Tawo, B.D.; Belli, H.L.L.; Proksch, P.; Tommy, D.; Hakim, E.H. A new antibacterial polyketide from the endophytic fungi *Aspergillus fumigatiaffinis*. *Nat. Prod. Commun.* **2018**, 13, 1573-1574.[10.1177/1934578X1801301202]
80. Zabala, A.O.; Xu, W.; Chooi, Y.H.; Tang, Y. Characterization of a silent azaphilone gene cluster from *Aspergillus niger* ATCC 1015 reveals a hydroxylation-mediated pyran-ring formation. *Chem. Biol.* **2012**, 19, 1049-1059. [10.1016/j.chembiol.2012.07.004]
81. de Oliveira Filho, J.W.; Islam, M.T.; Ali, E.S.; Uddin, S.J.; Santos, J.V.D.; de Alencar, M.V.O.B.; Gomes, A.L.; Paz, M.F.C.J.; de Brito, M.D.M.; Sousa, J.M.D.E.; et al. A comprehensive review on biological properties of citrinin. *Food Chem. Toxicol.* **2017**, 110, 130-141. [10.1016/j.fct.2017.10.002]
82. Guo, D.L.; Li, X.H.; Feng, D.; Jin, M.Y.; Cao, Y.M.; Cao, Z.X.; Gu, Y.C.; Geng, Z.; Deng, F.; Deng, Y. Novel polyketides produced by the endophytic fungus *Aspergillus fumigatus* from cordyceps sinensis. *Molecules* **2018**, 23,1709. [10.3390/molecules 23071709]
83. Song, Z.; Bakeer, W.; Marshall, J.W.; Yakasai, A.A.; Khalid, R.M.; Collemare, J.; Skellam, E.; Tharreau, D.; Lebrun, M.H.; Lazarus, C.M.; et al. Heterologous expression of the avirulence gene ACE1 from the fungal rice pathogen *Magnaporthe oryzae*. *Chem. Sci.* **2015**, 6, 4837-4845. [10.1039/c4sc03707c]
84. Grau, M.F.; Entwistle, R.; Chiang, Y.M.; Ahuja, M.; Oakley, C.E.; Akashi, T.; Wang, C.C.C.; Todd, R.B.; Oaldey, B.R.; et al. Hybrid transcription factor engineering activates the silent secondary metabolite gene cluster for (+)-asperlin in *Aspergillus nidulans*. *ACS Chem. Biol.* **2018**, 13, 3193-3205. [10.1021/acschembio.8b00679]
85. Wang, B.; Li, X.J.; Yu, D.; Chen, X.Y.; Tabudravu, J.; Deng, H.; Pan, L. Deletion of the epigenetic regulator GcnE in *Aspergillus* niger FGSC A1279 activates the production of multiple polyketide metabolites. *Microbiol. Res.* **2018**, 217, 101-107. [10.1016/j.micres.2018.10.004]
86. Yamazaki, H.; Takahashi, K.; Iwakura, N.; Abe, T.; Akaishi, M.; Chiba, S.; Namikoshi, M.; Uchida, R. A new protein tyrosine phosphatase 1B inhibitory alpha-pyrone-type polyketide from Okinawan plant-associated *Aspergillus* sp. TMPU1623. *J. Antibiot. (Tokyo)* **2018**, 71, 745-748. [10.1038/s41429-018-0054-y]
87. Siriwardane, A.M.; Kumar, N.S.; Jayasinghe, L.; Fujimoto, Y. Chemical investigation of metabolites produced by an endophytic *Aspergillus* sp. isolated from Limonia acidissima. *Nat. Prod. Res.* **2015**, 29, 1384-1387. [10.1080/14786419.2015.1025 230]
88. Du, L.; Zhu, T.J.; Fang, Y.C.; Liu, H.B.; Gu, Q.Q.; Zhu, W.M. Aspergiolide A, a novel anthraquinone derivative with naphtho[1,2,3-de]chromene-2,7-dione skeleton isolated from a marine-derived fungus *Aspergillus glaucus*. *Tetrahedron* **2007**, 63, 1085-1088. [10.1016/j.tet.2008.03.012]
89. Du, L.; Ai, J.; Li, D.H.; Zhu, T.J.; Wang, Y.; Knauer, M.; Bruhn, T.; Liu, H.B.; Geng, M.Y.; Gu, Q.Q.; et al. Aspergiolides C and D: spirocyclic aromatic polyketides with potent protein kinase c-Met inhibitory effects. *Chemistry* **2011**, 17, 1319-1326. [10.1002/chem.201001547]
90. Scherlach, K.; Sarkar, A.; Schroeckh, V.; Dahse, H.M.; Roth, M.; Brakhage, A.A.; Horn, U.; Hertweck, C. Two induced fungal polyketide pathways converge into antiproliferative spiroanthrones. *Chembiochem* **2011**, 12, 1836-1839. [10.1002/cbic.201100132]
91. Xie, L.; Tang, H.L.; Song, J.W.; Long, J.Y.; Zhang, L.L.; Li, X.F. Chrysophanol: a review of its pharmacology, toxicity and pharmacokinetics. *J. Pharm. Pharmacol.* **2019**, 71, 1475-1487. [10.1111/jphp.13143]
92. Paranjape, S.R.; Chiang, Y.M.; Sanchez, J.F.; Entwistle, R.; Wang, C.C.C.; Oakley, B.R.; Gamblin, T.C. Inhibition of tau aggregation by three *Aspergillus nidulans* secondary metabolites: 2,omega-dihydroxyemodin, asperthecin, and asperbenzal -dehyde. *Planta Med.* **2014**, 80, 77-85. [10.1055/s-0033-1360180]
93. Zhang, S.S.; Zhu, A. O.; Bai, X.; Zhu, H.J.; Cao, F. Alkaloids and polyketides from the marine-derived fungus *Aspergillus versicolor*. *Chem. Nat. Compd+.* **2020**, 56, 964-967. [10.1007/s10600-020-03203-y]
94. Frisvad, J.C.; Larsen, T.O. Extrolites of *Aspergillus fumigatus* and other pathogenic species in *Aspergillus* section fumigati. *Front Microbiol.* **2015**, 6, 1485. [10.3389/fmicb.2015.01485]
95. Hayashi, A.; Fujioka, S.; Nukina, M.; Kawano, T.; Shimada, A.; Kimura, Y. Fumiquinones A and B, nematicidal quinones produced by *Aspergillus fumigatus*. *Biosci. Biotechnol. Biochem.* **2007**, 71, 1697-1702. [10.1271/bbb.70110]
96. Kong, C.X.; Huang, H.Z.; Xue, Y.; Liu, Y.Q.; Peng, Q.Q.; Liu, Q.; Xu, Q.; Zhu, Q.Y.; Yin, Y.; Zhou, X.S.; et al. Heterologous pathway assembly reveals molecular steps of fungal terreic acid biosynthesis. *Sci. Rep.* **2018**, 8, 2116. [10.1038/s41598-018- 20514-x]

97. Seshime, Y.; Juvvadi, P.R.; Kitamoto, K.; Ebizuka, Y.; Fujii, I. Identification of csypyrone B1 as the novel product of *Aspergillus oryzae* type III polyketide synthase CsyB. *Bioorg. Med. Chem.* **2010**, 18, 4542-4546. [10.1016/j.bmc.2010.04.058]
98. Xu, Z.; Xiong, B.; Xu, J. Chemical investigation of secondary metabolites produced by mangrove endophytic fungus phyllosticta capitalensis. *Nat. Prod. Res.* **2021**, 35,1561-1565. [10.1080/14786419.2019.1656624]
99. Chobot, V.; Opletal, L.; Jahodar, L.; Patel, A.V.; Dacke, C.G.; Blunden, G. Ergosta-4,6,8,22-tetraen-3-one from the edible fungus, *Pleurotus ostreatus* (oyster fungus). *Phytochemistry* **1997**,45, 1669-1671. [10.1016/S0031-9422(97)00249-5]
100. Lee, D.Y.; Lee, S.J.; Kwak, H.Y.; Lakoon-Jung.; Heo, J.; Hong, S.; Kim, G.W.; Baek, N.I. Sterols isolated from Nuruk (*Rhizopus oryzae* KSD-815) inhibit the migration of cancer cells. *J. Microbiol. Biotechn.* **2009**,19, 1328-1332. [10.4014/jmb.0902.0072]
101. Salendra, L.; Lin, X.P.; Chen, W.H.; Pang, X.Y.; Luo, X.W.; Long, J.Y.; Liao, S.R.; Wang, J.F.; Zhou, X.F.; Liu, Y.H. Cytotoxicity of polyketides and steroids isolated from the sponge-associated fungus *Penicillium citrinum* SCSIO 41017. *Nat. Prod. Res.* **2021**,35, 900-908. [10.1080/14786419.2019.1610757]
102. Springer, J.P.; Dorner, J.W.; Cole, R.J.; Cox, R.H. Terretonin, a toxic compound from *Aspergillus terreus*. *J. Org. Chem.* **1979**, 44, 4852-4854.[10.1021/jo00394a023]
103. Konig, C.C.; Scherlach, K.; Schroeckh, V.; Horn, F.; Nietzsche, S.; Brakhage, A.A.; Hertweck, C. Bacterium induces cryptic meroterpenoid pathway in the pathogenic fungus *Aspergillus fumigatus*. *Chembiochem* **2013**,14, 938-942. [10.1002/cbic.201300070]
104. Hamasaki, T.; Kuwano, H.; Isono, K.; Hatsuda, Y.; Fukuyama, K.; Tsukihara, T.; Katsube, Y. A new metabolite, parasiticolide A, from *Aspergillus parasiticus*. *Agric. Biol. Chem.* **2014**,39, 749-751.[10.1080/00021369.1975.10861681]
105. Hu, Z.X.; Sun, W.G.; Li, Q.; Li, X.N.; Zhu, H.C.; Huang, J.F.; Liu, J.J.; Wang, J.P.; Xue, Y.B.; Zhang, Y.H. Spiroaspertrione A, a bridged spirocyclic meroterpenoid, as a potent potentiator of oxacillin against methicillin-resistant staphylococcus aureus from *Aspergillus* sp. TJ23. *J. Org. Chem.* **2017**,82, 3125-3131. [10.1021/acs.joc.7b00056]
106. Qiao, Y.B.; Zhang, X.T.; He, Y.; Sun, W.G.; Feng, W.Y.; Liu, J.J.; Hu, Z.X.; Xu, Q.Q.; Zhu, H.C.; Zhang, J.W.; et al. Aspermerodione, a novel fungal metabolite with an unusual 2,6-dioxabicyclo[2.2.1]heptane skeleton, as an inhibitor of penicillin-binding protein 2a. *Sci. Rep.* **2018**, 8, 5454. [10.1038/s41598-018-23817-1]
107. Yan, T.; Guo, Z.K.; Jiang, R.; Wei, W.; Wang, T.; Guo, Y.; Song, Y.C.; Tan, R.X.; Ge, H.M. New flavonol and diterpenoids from the endophytic fungus *Aspergillus* sp. YXf3. *Planta Med.* **2013**, 79, 348-352. [10.1055/s-0032-1328260]
108. Hawas, U.W.; El-Beih, A.A.; El-Halawany, A.M. Bioactive anthraquinones from endophytic fungus *Aspergillus versicolor* isolated from red sea algae. *Arch. Pharm. Res.* **2012**, 35, 1749-1756. [10.1007/s12272-012-1006-x]
109. Uchoa, P.K.S.; Pimenta, A.T.A.; Braz, R.; de Oliveira, M.D.F.; Saraiva, N.N.; Rodrigues, B.S.F.; Pfenning, L.H.; Abreu, L.M.; Wilke, D.V.; Florenciog, K.G.D.; et al. New cytotoxic furan from the marine sediment-derived fungi *Aspergillus niger*. *Nat. Prod. Res.* **2017**, 31, 2599-2603. [10.1080/14786419.2017.1283499]
110. Pornpakakul, S.; Liangsakul, J.; Ngamrojanavanich, N.; Roengsumran, S.; Silhanonth, P.; Piapukiew, J.; Sangvichien, E.; Puthong, S.; Petsom, A. Cytotoxic activity of four xanthones from *Emericella variecolor*, an endophytic fungus isolated from Croton oblongifolius. *Arch. Pharm. Res.* **2006**, 29, 140-144. [10.1007/BF02974275]
111. Andersen, R.; Andersen, R.; Buchi, G.; Kobbe, B.; Demain, A.L. Secalonic acids D and F are toxic metabolites of *Aspergillus aculeatus*. *J. Org. Chem.* **1977**, 42, 352-353. [10.1021/jo00422a042]
112. Alberts, A.W.; Alberts, A.W.; Chen, J.; Kuron, G.; Hunt, V.; Huff, J.; Hoffman, C.; Rothrock, J.; Lopez, M.; Joshua, H.; Harris, E.; et al. Mevinolin: a highly potent competitive inhibitor of hydroxymethylglutaryl-coenzyme a reductase and a cholesterol -lowering agent. *Proc. Nat.l Acad. Sci. U S A.* **1980**,77, 3957-3961. [10.1073/pnas.77.7.3957]
113. Tsukamoto, S.; Miura, S.; Yamashita Y.; Ohta T. Aspermytin A: a new neurotrophic polyketide isolated from a marine -derived fungus of the genus *Aspergillus*. *Bioorg. Med. Chem. Lett.* **2004**,14, 417-420.[10.1016/j.bmcl.2003.10.053]
114. Zhuravleva, O.I.; Afiyatullov, S.S.; Vishchuk, O.S.; Denisenko, V.A.; Slinkina, N.N.; Smetanina, O.F. Decumbenone C, a new cytotoxic decaline derivative from the marine fungus *Aspergillus sulphureus* KMM 4640. *Arch. Pharm. Res.* **2012**,35, 1757-1762. [10.1007/s12272-012-1007-9]

115. Zhuravleva, O.I.; Kirichuk, N.N.; Denisenko, V.A.; Dmitrenok, P.S.; Pivkin, M.V.; Afiyatullov, S.S. New kipukasin from marine isolate of the fungus *Aspergillus flavus*. *Chem. Nat. Compd+.* **2016**, 52, 266-268. [10.1007/s10600-016-1610-y]
116. Petersen, L.M.; Hoeck, C.; Frisvad, J.C.; Gotfredsen, C.H.; Larsen, T.O. Dereplication guided discovery of secondary metabolites of mixed biosynthetic origin from *Aspergillus aculeatus*. *Molecules* **2014**,19, 10898-10921. [10.3390/molecules190 810898]
117. Jackson, M.; Karwowski, J.P.; Humphrey, P.E.; Kohl, W.L.; Barlow, G.J.; Tanaka, S.K. Calbistrins, novel antifungal agents produced by *Penicillium restrictum*. I. Production, taxonomy of the producing organism and biological activity. *J. Antibiot. (Tokyo)* **1993**,46, 34-38. [10.7164/antibiotics.46.34]
118. Fu, Y.; Wu, P.; Xue, J.H.; Wei, X.Y.; Li, H.X. Versicorin, a new lovastatin analogue from the fungus *Aspergillus versicolor* SC0156. *Nat. Prod. Res.* **2015**, 29, 1363-1368. [10.1080/14786419.2015.1026342]
119. Tsukamoto, S.; Yoshida, T.; Hosono, H.; Ohta, T.; Yokosawa, H. Hexylitaconic acid: a new inhibitor of p53-HDM2 interaction isolated from a marine-derived fungus, *Arthrinium* sp. *Bioorg. Med. Chem. Lett.* **2006**, 16, 69-71. [10.1016/j.bmcl.2005.09.052]
120. Kaaniche, F.; Hamed, A.; Abdel-Razek, A.S.; Wibberg, D.; Abdissa, N.; El Euch, I.Z.; Allouche, N.; Mellouli, L.; Shaaban, M.; Sewald, N. Bioactive secondary metabolites from new endophytic fungus *Curvularia* sp. isolated from Rauwolfia macrophylla. *PLoS One* **2019**, 14, e0217627. [10.1371/journal.pone.0217627]
121. Arakawa, M.; Someno, T.; Kawada, M.; Ikeda, D. A new terrein glucoside, a novel inhibitor of angiogenin secretion in tumor angiogenesis. *J. Antibiot. (Tokyo)* **2008**, 61, 442-448. [10.1038/ja.2008.60]
122. Singh, S.B.; Jayasuriya, H.; Zink, D.L.; Polishook, J.D.; Dombrowski, A.W.; Zweerink, H. Aspercyclide A–C, three novel fungal metabolites from *Aspergillus* sp. as inhibitors of high-affinity IgE receptor. *Tetrahedron Lett.* **2004**, 45, 7605-7608. [10.1016/j.tetlet.2004.08.116]
123. Xu, Y.Q.; Espinosa-Artiles, P.; Schubert, V.; Xu, Y.M.; Zhang, W.; Lin, M.; Gunatilaka, A.A.L.; Sussmuth, R.; Molnar, I. Characterization of the biosynthetic genes for 10,11-dehydrocurvularin, a heat shock response-modulating anticancer fungal polyketide from *Aspergillus terreus*. *Appl. Environ. Microb.* **2013**, 79, 2038-2047. [10.1128/AEM.03334-12]
124. Santagata, S.; Xu, Y.M.; Wijeratne, E.M., Kontnik, R.; Rooney, C.; Perley, C.C.; Kwon, H.; Clardy, J.; Kesari, S.; Whitesell, L.; Lindquist, S.; Gunatilaka, A.A.L. Using the heat-shock response to discover anticancer compounds that target protein homeostasis. *ACS Chem. Biol.* **2012,** 7, 340-349. [10.1021/cb200353m]
